# Comparison of Two Chemotherapy Regimens After First-Line Treatment for HER2-Negative Metastatic Gastric Cancer

**DOI:** 10.7759/cureus.38837

**Published:** 2023-05-10

**Authors:** Zuhat Urakçı, Senar Ebinç, Sezai Tunç, Ziya Kalkan, Zeynep Oruç, Mehmet Küçüköner, Muhammet Ali Kaplan, Abdurrahman Isikdogan

**Affiliations:** 1 Department of Medical Oncology, Dicle University Faculty of Medicine, Diyarbakır, TUR; 2 Department of Medical Oncology, Gazi Yasargil Training and Research Hospital, Diyarbakır, TUR

**Keywords:** comparison of chemotherapy, subsequent-line treatments, paclitaxel, folfiri, gastric cancer

## Abstract

Aim: Metastatic stage gastric cancer is a disease with a poor prognosis and the likelihood of achieving a cure in these patients is low. Treatment response to subsequent-line treatments is poor. We aimed to investigate the effectiveness of the folinic acid, fluorouracil and irinotecan (FOLFIRI) and paclitaxel+carboplatin regimens, which are used in subsequent lines of therapy in advanced-stage gastric cancer.

Materials and methods: This study included 40 patients who have metastatic stage gastric cancer and received FOLFIRI or paclitaxel+carboplatin therapy in subsequent lines of therapy between 2017 and 2022. The data of the patients were analyzed retrospectively.

Results: At diagnosis median age was 51 (23-88) years. The tumor was localized in the gastroesophageal junction in eight (20%) patients and in other gastric locations in 32 (80%) patients. At diagnosis, 75% (n=30) of the patients presented with the disease in the metastatic stage, while 25% (n=10) presented with stage II-III disease. Regarding the treatments received in the second and further lines of therapy, 18 (45%) patients received paclitaxel+carboplatin and 22 (55%) patients received a FOLFIRI regimen. Of these treatments, 67.5% (n=27) were given as the second line and 32.5% (n=13) were given as third-line therapy. The objective response rate (ORR) was 45.5% in the FOLFIRI arm compared to 16.7% in the paclitaxel+carboplatin arm (p=0.05). Both treatment arms had a median progression-free survival (PFS) of three months (p=0.82). The median overall survival (OS) time was seven months in the FOLFIRI arm compared to eight months in the paclitaxel+carboplatin arm (p=0.71). Side effects were similar between both treatment arms.

Conclusion: This study determined that FOLFIRI and paclitaxel+carboplatin treatments have similar OS, PFS, and side effect profiles in subsequent line treatment of gastric cancer. The FOLFIRI treatment regimen yielded a higher ORR.

## Introduction

Locally advanced unresectable or metastatic-stage gastric and gastroesophageal cancers are lethal diseases and the probability of achieving a cure in these patients is low. Most of the time, the purpose of the treatment is to offer palliation and increase survival. Symptom palliation and cytotoxic treatments are known to offer a survival advantage in advanced-stage disease. Currently, the standard of care in advanced disease is chemotherapy based on fluoropyrimidine and platin combination with immunotherapy based on Checkmate 649 trial [1]. Previous studies have shown that administering cytotoxic chemotherapy to patients with appropriate performance status after first-line therapy contributes to survival when compared with best supportive care (BSC) [2]. However, nearly half of the patients fail to achieve treatment response [3]. From previous phase-3 studies; the AIO study compared irinotecan, the COUGAR study compared docetaxel, the REGARD study compared ramucirumab, and the RAINBOW study compared ramucirumab+paclitaxel with BSC, where the chemotherapy arms were shown to offer a survival advantage [4-7]. There is no detailed data regarding the comparison of the folinic acid, fluorouracil and irinotecan (FOLFIRI) and paclitaxel+carboplatin regimens in the first and further lines of therapy in the treatment of gastric cancer. In this study, we aimed to compare irinotecan- (FOLFIRI) and taxane-based (paclitaxel+carboplatin) therapies, which are among the post-first-line treatments used in HER2-negative advanced-stage gastric cancer.

## Materials and methods

This study included 40 patients who presented to our oncology center with a diagnosis of metastatic stage gastric cancer and received FOLFIRI or paclitaxel+carboplatin treatment in subsequent lines of therapy due to their diagnoses between 2017 and 2022. Patient data regarding age, gender, performance status, tumor subtype, disease stage at diagnosis, received treatments, treatment response status, and survival were recorded. Patients with HER2 amplification were not included in the study.

Definitions and treatments

The staging was performed according to the American Joint Committee on Cancer (AJCC) version 8 TNM staging system. Performance status was determined based on the Eastern Cooperative Oncology Group (ECOG) criteria. The patients were given 80 mg/m^2^ paclitaxel (days 1, 8, 15) + carboplatin area under the curve (AUC) 4-5 (day 1) every 21 days until progression or FOLFIRI (400 mg/m^2^ folinic acid (day 1) + 400 mg/m^2^ 5-Fluorouracil IV bolus (day 1) + 2400-3000 mg/m^2^ 5-Fluorouracil (46-hour infusion) + 180mg/m^2^ irinotecan (day 1)) every 14 days until progression as second or third line therapy. Response was evaluated every three months or earlier based on the clinical status, by computerized tomography or positron emission tomography according to RECIST (Response Evaluation Criteria in Solid Tumors) v 1.1. The sum of the partial and complete response rates at the third month of treatment was calculated as the objective response rate (ORR). Progression-free survival (PFS) was calculated as the duration of time from treatment initiation to progression, and metastatic overall survival (mOS) was calculated as the duration of time from the occurrence of metastasis to death. Overall survival (OS) for the treatment arms was accepted as the duration of time from treatment initiation to death.

Statistical analysis

For statistical analysis of the study, the PASW Statistics for Windows, Version 18.0 (IBM SPSS Statistics, Armonk, NY) was used. Descriptive statistics were used for patient characteristics and frequency of parameters. Fisher's exact, chi-square and student's t-tests were used to evaluate the distribution between groups. Univariate and multivariate assessments of survivals were analyzed by Cox regression analysis. Kaplan-Meier survival analysis was used for the survival times. The p-significance value was accepted as <0.05.

Ethical approval

It was obtained from the Dicle University Faculty of Medicine Ethics Committee (date/number: 12.12.2022/18).

## Results

A total of 40 patients, 13 (32.5%) female and 27 (67.5%) male were included in our study. The median patient age at diagnosis was 51 (23-88) years. The most common histopathological subtype was diffuse-type adenocarcinoma with a rate of 72.5% (n=29). Regarding tumor localization; the tumor was localized in the gastroesophageal junction in eight (20%) patients and in other gastric locations in 32 (80%) patients. Of the patients, 75% (n=30) had the disease in the metastatic stage at diagnosis, while 25% (n=10) presented with stage II-III disease. Eleven (27.5%) patients had undergone surgery, four (10%) had received radiotherapy, and 27 (67.5%) patients had received adjuvant or neoadjuvant therapy. Regarding the treatments given in the second and further lines of therapy, 18 (45%) patients had received paclitaxel+carboplatin and 22 (55%) patients had received the FOLFIRI regimen. Of these treatments, 67.5% (n=27) were used in second-line treatment and 32.5% (n=13) were used in third-line treatment (Table 1 and Table 2). The two treatment arms were not different in terms of patient clinicopathological characteristics (Table 2).

**Table 1:** General characterıstıc features of the patients FOLFIRI: Folinic acid, fluorouracil and irinotecan, ECOG PS: Eastern Cooperative Oncology Group performance status, GEJ: gastroesophageal junction

	N=40 (%)
Age year (median, range)	51 (23-88)
Gender	
Male	27 (67.5)
Female	13 (32.5)
ECOG PS	
0-1	31 (77.5)
≥ 2	9 (22.5)
Histological subtypes	
Intestinal type	4 (10)
Diffuse type	29 (72.5)
Other types	7 (17.5)
Tumor localization	
GEJ	8 (20)
Gastric	32 (80)
Stage at diagnosis	
II	1 (2.5)
III	9 (22.5)
IV	30 (75)
Surgery history	
Yes	11 (27.5)
No	29 (72.5)
Treatment line	
Second line	27 (67.5)
Third line	13 (32.5)
Chemotherapy regimen	
Paclitaxel + Carboplatin	18 (45)
FOLFIRI	22 (55)
Radiation therapy	
Yes	4 (10)
No	36 (90)

**Table 2:** Distribution of patients according to the chemotherapy regimen received *T-test, **Chi-square test, ***Fishers exact test, FOLFIRI: Folinic acid, fluorouracil and irinotecan, ECOG PS: Eastern Cooperative Oncology Group performance status, P + C: Paclitaxel + Carboplatin, GEJ: gastroesophageal junction

	All patients, n (%)	FOLFIRI	P + C	P value
Age year (mean, std. dev)	51.5 (±15.6)	50.7 (±15.7)	52.5 (±15.9)	0.71*
Gender				0.20**
Male	27 (67.5)	13 (59.1)	14 (77.8)	
Female	13 (32.5)	9 (40.9)	4 (22.2)	
ECOG PS				0.47***
0-1	31 (77.5)	16 (72.7)	15 (83.3)	
≥ 2	9 (22.5)	6 (27.3)	3 (16.7)	
Histological subtypes				1.00***
Diffuse type	29 (72.5)	16 (72.7)	13 (72.2)	
Other types	11 (27.5)	6 (27.3)	5 (27.8)	
Stage at diagnosis				1.00***
II-III	10 (25)	6 (27.3)	4 (22.2)	
IV	30 (75)	16 (72.7)	14 (77.8)	
Neo/adjuvant treatment				0.91**
Yes	27 (67.5)	7 (31.8)	6 (33.3)	
No	13 (32.5)	15 (68.2)	12 (66.7)	
Tumor localization				1.00***
GEJ	8 (20)	4 (18.2)	4 (22.2)	
Gastric	32 (80)	18 (81.8)	14 (77.8)	
Radiation therapy				0.61***
Yes	4 (10)	3 (13.6)	1 (5.6)	
No	36 (90)	19 (86.4)	17 (94.4)	
Treatment line				0.56**
Second line	27 (67.5)	14 (63.6)	13 (72.2)	
Third line	13 (32.5)	8 (36.4)	5 (27.8)	

Regarding patient response at three months, patients receiving FOLFIRI showed a partial response at a rate of 45.5%, stable disease at a rate of 4.5%, and progression at a rate of 50%, while the paclitaxel+carboplatin arm showed a partial response at a rate of 16.7%, stable disease at a rate of 27.8%, and progression at a rate of 55.6%. Neither arm had patients who achieved complete response. The ORR was 45.5% in the FOLFIRI arm compared to 16.7% in the paclitaxel+carboplatin arm (p=0.05). The ORR was significantly higher in the FOLFIRI arm compared to the paclitaxel+carboplatin arm (p=0.05) (Table 3).

**Table 3:** Response rates and survival outcomes FOLFIRI: Folinic acid, fluorouracil and irinotecan, CR: complete response, PR: partial response, SD: stable disease, PD: progressive disease, ORR: objective response rate, PFS: progression-free survival, mo: months

	FOLFIRI	Paclitaxel + Carboplatin	P value
Response (n, %)			
CR	0 (0)	0 (0)	
PR	10 (45.5)	3 (16.7)	
SD	1 (4.5)	5 (27.8)	
PD	11 (50)	10 (55.6)	
ORR	10 (45.5)	3 (16.7)	0.05
PFS (mo)	3	3	0.82
PFS for 2nd line	3	3	0.63
PFS for 3rd line	2	2	0.48

**Figure 1:**
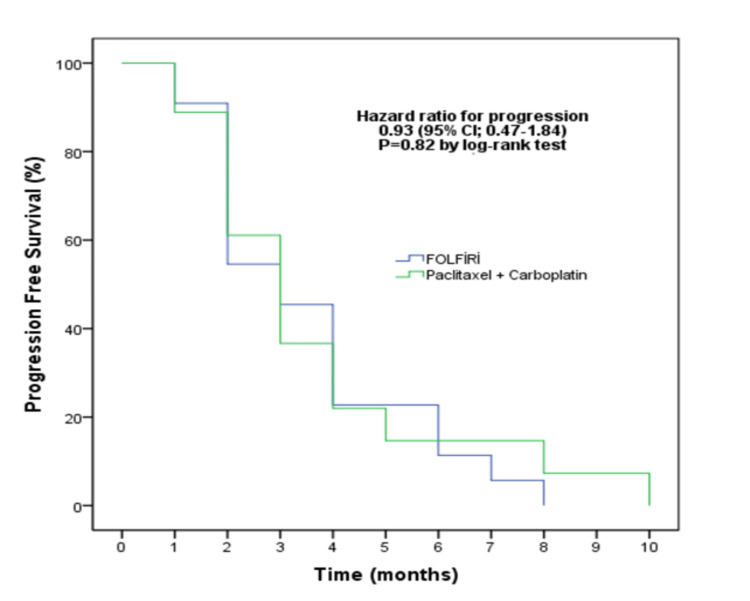
Comparison of treatment arms in terms of progression-free survival

The median PFS time was three months in both treatment arms (p=0.82) (Figure 1).

**Figure 2:**
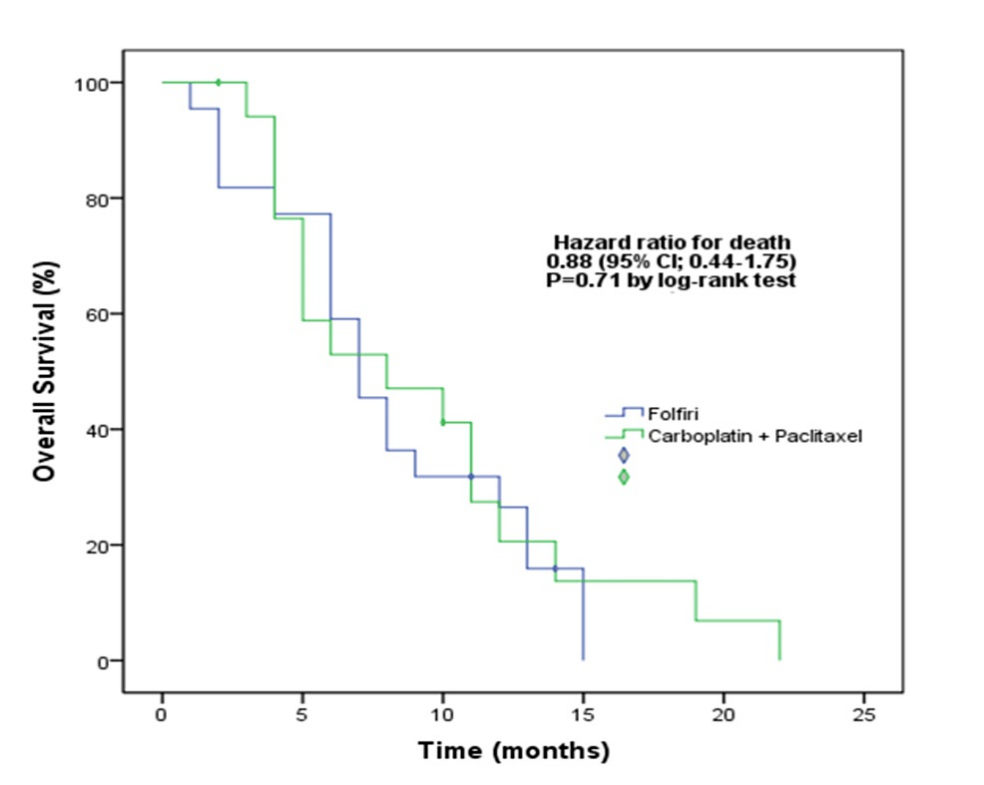
Comparison of treatment arms in terms of overall survival

The median OS from the occurrence of metastatic disease was calculated as 16 months for both the FOLFIRI and the paclitaxel+carboplatin arm (hazard ratio (HR): 0.94, 95% confidence interval (CI): 0.48-1.81, Log-rank p=0.84). The median OS from the initiation of the second-line therapy was seven months in the FOLFIRI arm and eight months in the paclitaxel+carboplatin arm. Both groups had similar OS results (HR: 0.88, 95% CI: 0.44-1.75, Log-rank p=0.71) (Figure 2).

The evaluation of the parameters that could potentially influence PFS (age, gender, Eastern Cooperative Oncology Group performance status (ECOG PS), histological subtype, receipt of neoadjuvant/adjuvant treatment, tumor localization, radiotherapy and chemotherapy regimens, line of treatment) in multivariate analyses determined that history of radiotherapy (HR: 3.5, 95% CI: 1.09-11.16, p=0.03) and receipt of the treatment regimens (FOLFIRI/paclitaxel+carboplatin) as third-line therapy (HR: 2.45, 95% CI: 1.14-5.26, p=0.02) were associated with a significantly shorter PFS (Table 4).

**Table 4:** Univariate and multivariate analysis results for progression-free survival FOLFIRI: Folinic acid, fluorouracil and irinotecan, ECOG PS: Eastern Cooperative Oncology Group performance status, P + C: Paclitaxel + Carboplatin, GEJ: gastroesophageal junction, HR: hazard ratio, CI: confidence interval, * reference category

	Univariate analysis			Multivariate analysis			
	HR	95% CI	P		HR	95% CI	P
Age	0.98	0.96-1.00	0.10				
Gender (male*/female)	0.98	0.49-1.99	0.97				
ECOG PS (0-1*/≥ 2)	0.68	0.29-1.62	0.39				
Histological subtypes (diffuse*/others)	0.52	0.23-1.15	0.11		0.45	0.19-1.07	0.07
Neo/adjuvant treatment (no*/yes)	0.90	0.44-1.84	0.78				
Tumor localization (GEJ*/gastric)	1.00	0.41-2.43	1.00				
Radiation therapy (no*/yes)	3.22	1.05-9.89	0.04		3.5	1.09-11.16	0.03
Chemotherapy regimen (FOLFIRI*/P + C)	0.93	0.47-1.84	0.85				
Treatment line (2nd*/3rd)	1.82	0.88-3.74	0.10		2.45	1.14-5.26	0.02

From histological subtypes, diffuse adenocarcinoma was associated with a poorer PFS, which approached the threshold of statistical significance (HR: 0.45, 95% CI: 0.19-1.07, p=0.07). The chemotherapy regimens (FOLFIRI/paclitaxel+carboplatin) were not significantly different in terms of PFS (HR: 0.93, 95% CI: 0.47-1.84, p=0.85) (Table 4).

The most common chemotherapy-induced side effects included anemia, neutropenia, and nausea-vomiting in both groups. The rates of grade 3-4 thrombocytopenia, anemia, neutropenia, and febrile neutropenia were 13.6%, 40.9%, 22.7%, and 4.5% in the FOLFIRI group compared to 5.6%, 50%, 11.1%, and 16.7% in the paclitaxel+carboplatin arm, respectively. The two treatment arms were not significantly different with regard to these side effects. The rate of nausea-vomiting regardless of grade was 86.4% in the FOLFIRI and 83.3% in the paclitaxel+carboplatin arm (p=1.00). Other side effects including hepatotoxicity, nephrotoxicity, and mucositis had comparable rates between the two groups (Table 5).

**Table 5:** Frequency of side effects according to chemotherapy regimens * Chi-square test, **Fishers exact test, a for grades 3-4, b for any grade, FOLFIRI: Folinic acid, fluorouracil and irinotecan

	FOLFIRI		Paclitaxel + Carboplatin		
	Any grade	Grades 3-4	Any grade	Grades 3-4	P
Anemia	21 (95.4)	9 (40.9)	15 (83.3)	9 (50)	0.56* ^(a)^
Neutropenia	21 (95.4)	5 (22.7)	15 (83.3)	2 (11.1)	0.42** ^(a)^
Thrombocytopenia	8 (36.3)	3 (13.6)	6 (33.3)	1 (5.6)	0.61** ^(a)^
Nephrotoxicity	1 (4.5)	0 (0)	0 (0)	0 (0)	1.00** ^(b)^
Hepatotoxicity	1 (4.5)	0 (0)	1 (5.6)	0 (0)	1.00** ^(b)^
Mucositis	4 (18.2)	0 (0)	1 (5.6)	0 (0)	0.35** ^(b)^
Nausea-vomiting	19 (86.4)	0 (0)	15 (83.3)	0 (0)	1.00** ^(b)^
Febrile neutropenia	1 (4.5)		3 (16.7)		0.31** ^(b)^

## Discussion

Advanced-stage gastric cancer is a disease associated with a highly unfavorable prognosis and a very low rate of cure. Response rates and the likelihood of response further decline in the second and further lines of therapy. However, despite the new studies in the field of treatment, chemotherapy is still recommended to treat refractory metastatic gastric cancer patients. The comparison of salvage chemotherapy options versus BSC revealed a survival benefit. A phase III randomized clinical study determined an OS time of 5.3 months with salvage chemotherapy+BSC compared to 3.8 months in patients followed up with BSC alone. The mentioned study identified age, performance status, hemoglobin level, number of treatments received, duration of response to previous treatments, and number of metastatic sites as prognostic factors. In the same study, docetaxel and irinotecan yielded similar OS outcomes (5.2 vs 6.5 months, p=0.116) [2]. Specifically, the AIO study compared irinotecan and BSC in the second-line treatment of advanced-stage gastric cancer. The OS was four months in the irinotecan arm in comparison to two months in the BSC arm (p=0.012) [4]. The COUGAR-02 study randomized 168 treatment-refractory patients with gastroesophageal adenocarcinoma into active symptom control and docetaxel. This study determined the median OS as 5.2 months in the docetaxel arm compared to 3.6 months in the supportive care arm (p=0.01) and emphasized that docetaxel could be a second-line treatment option in gastroesophageal cancer [5]. In the REGARD study, ramucirumab, which is a VEGFR-2 antagonist, that was compared to a placebo. Ramucirumab achieved an OS of 5.2 months, while the placebo resulted in an OS of 3.8 months. Thus, a survival benefit was shown for ramucirumab in the second-line treatment for gastric cancer [6]. Ramucirumab is not among the agents that are widely used in our country as it is not included in the national reimbursement program. In the multicenter, double-blind, randomized phase-III RAINBOW study, paclitaxel was added to ramucirumab therapy and was compared to placebo+paclitaxel in previously treated advanced-stage gastric or GEJ adenocarcinoma. This study determined a median OS of 9.6 months for the ramucirumab+paclitaxel arm compared to 7.4 months for the placebo + paclitaxel arm (p=0.017). Thus, adding ramucirumab to chemotherapy in advanced gastric and GEJ adenocarcinoma was shown to be beneficial [7]. In a phase-II study comparing combination chemotherapy with monotherapy, single-agent irinotecan was compared with mFOLFIRI in advanced-stage gastric adenocarcinoma. This study determined an ORR of 17.2% in the irinotecan arm compared to 20% in the mFOLFIRI arm (p=0.525), and a PFS of 2.2 months in the irinotecan arm compared to 3.0 months in the mFOLFIRI arm (p=0.481). The two arms offered comparable response and survival rates. OS was 5.8 months and 6.7 months in the irinotecan and mFOLFIRI arms, respectively (p=0.514) [8]. In a phase-III study comparison of combination therapy and monotherapy, irinotecan+platin treatment was superior to irinotecan monotherapy. This study showed a median PFS of 3.8 months in the combination arm compared to 2.8 months in the monotherapy arm (p=0.039) [9]. In a similar design, the TRICS phase-III study compared irinotecan+cisplatin combination therapy with irinotecan monotherapy in advanced gastric cancer that recurred or progressed after S1 monotherapy. In contrast with the previous study, this study did not determine a difference between combination therapy and monotherapy in terms of OS (13.9 months vs 12.7 months, p=0.288) or PFS (4.6 months vs 4.1 months, p=0.376). However, subgroup analyses suggested that the combination therapy contributed to OS in intestinal-type histology (15.8 vs 14 months, p=0.019) [10]. In a phase-II study on the FOLFIRI treatment regimen as combination chemotherapy in previously treated metastatic stage gastric cancer, the response rate was 18.2%, the disease control rate was 36%, the median OS and median time to progression was 5.1 months and 2.3 months, respectively [11]. In our study, the median OS was calculated as seven months for the FOLFIRI arm. Median PFS times were three months and two months for patients receiving FOLFIRI as second-line treatment and third-line treatment, respectively. Our results regarding this treatment arm were consistent with the outcomes of irinotecan-based treatments reported in the literature. In our study, the ORR to FOLFIRI was 45.5%, exceeding the rates reported in the literature. This may be attributed to differences in patient characteristics. In a retrospective study evaluating weekly paclitaxel therapy, the median survival time was determined as 151 days and it was stated that paclitaxel could be an appropriate option in second or further lines of therapy in advanced-stage gastric cancer [12]. A phase-II study that evaluated tri-weekly paclitaxel as second-line therapy in advanced-stage gastric cancer determined a median OS of eight months. It was suggested that tri-weekly paclitaxel could be administered as salvage therapy in advanced-stage gastric cancer [13]. In a phase-II study, an ORR of 27.7% was reported with weekly paclitaxel+carboplatin combination therapy in 47 previously treated gastric cancer patients [14]. In another phase-II study, paclitaxel+carboplatin resulted in an ORR of 22% and an OS of 32 weeks in gastric cancer patients previously treated with fluorouracil and platin [15]. In our study, the median PFS was three months and two months in patients receiving paclitaxel+carboplatin as second-line and third-line therapy, respectively. The ORR was 16.7% and the median OS was eight months. Although our ORR outcomes were moderately lower than those reported in the literature, our PFS and OS outcomes were consistent with the literature. The lower response rate in our study may be attributable to the use of paclitaxel+carboplatin as a third-line treatment in some of our patients. In an open-label phase-III randomized study comparing paclitaxel and irinotecan, which are two agents used in the second-line treatment of advanced-stage gastric cancer, the two treatment options were found to be similar in terms of OS and PFS. In this study, irinotecan and paclitaxel were reported to yield OS times of 9.5 months versus 8.4 months (p=0.38) and PFS times of 3.6 months versus 2.8 months (p=0.33), respectively [16]. The literature does not contain any comprehensive studies comparing the paclitaxel+carboplatin and FOLFIRI combination regimens. Our study compared the paclitaxel+carboplatin and FOLFIRI regimens, which are combination therapies used in second and further lines of therapy in advanced-stage gastric cancer. Regarding response rates, the ORR was 45.5% with FOLFIRI in comparison to 16.7% in the paclitaxel+carboplatin arm. Most of the responses obtained in the FOLFIRI arm were partial responses (45.5%), while paclitaxel+carboplatin more commonly resulted in stable disease as the response (27.8%) (Table 3). The ORR was higher in the FOLFIRI arm when compared with the paclitaxel+carboplatin arm with statistical significance. In both treatment arms, half of the patients showed progression at the first evaluation. In survival analysis, mOS was found at 16 months for both treatment arms (p=0.85). OS from the initiation of second-line treatment was seven months in the FOLFIRI arm and eight months in the paclitaxel+carboplatin arm. The OS outcomes of the two groups were statistically similar (HR: 0.88, 95% CI: 0.44-1.75, p=0.71). When evaluated with regard to PFS, both treatment arms yielded a median PFS of three months (HR: 0.93, 95% CI: 0.47-1.84, p=0.82). PFS outcomes were similar between the two treatment arms.

Neutropenia takes precedence as a common toxicity encountered in patients with advanced-stage gastric cancer receiving FOLFIRI. Accordingly, the most commonly reported grade 3-4 side effect in our study was neutropenia. Previous studies have reported rates of grade 3-4 neutropenia varying between 10 and 40% [11,17-19]. In our study, the rate of grade 3-4 neutropenia was 22.7%, which is consistent with the rates reported in the literature. A rate of grade 3-4 neutropenia approaching 30% was reported with the use of paclitaxel as second-line treatment in advanced-stage gastric cancer [20]. In our study, the rate of grade 3-4 neutropenia was 11% in the paclitaxel+carboplatin arm. This rate was lower than those reported in the literature. In our study, the most common grade 3-4 side effect was anemia in both treatment arms. Since gastric cancer has a high risk of bleeding, this may be attributed to gastrointestinal tract hemorrhage rather than a treatment side effect. In our study, the two treatment arms were found to have similar side effect profiles in terms of hematological, renal, hepatic, and mucositis toxicities (Table 5). In evaluating these, it should be noted that the retrospective design of the study might entail problems with the reporting of side effects.

The limitations of our study include its retrospective, single-center design, as well as the heterogeneity of the treatment groups and the low number of patients.

## Conclusions

This study observed similar OS, PFS, and side effect profiles for the FOLFIRI and paclitaxel+carboplatin treatments. However, a higher ORR was obtained with the FOLFIRI treatment regimen, with the difference approaching the threshold of statistical significance (p=0.05).
